# Supplementary material for: Infant Feeding Pattern Clusters Are Associated with Childhood Health Outcomes
Source: Nutrients. 2023 Jul 7;15(13):3065. doi: 10.3390/nu15133065 (PMC10346276; doi:10.3390/nu15133065)
Supplement: Supplementary file 1 [file nutrients-15-03065-s001.zip › nutrients-2477615-supplementary.pdf]

Supplementary Table S1. Definitions of childhood diseases

**Disease**	**Definitions**
Upper respiratory tract disorders	
Adenotonsillectomy	Procedure of tonsillectomy or adenoidectomy[1]
Chronic otitis media	A principal diagnosis of ICD-10 H65.X–H67.X and procedure of myringotomy or insertion of ventilation tube
Hospitalization for croup	Hospitalization with a principal diagnosis of ICD-10 code (J04.X–J05.X)[2]
Hospitalization for URI	Hospitalization with a principal diagnosis of ICD-10 codes (J00.X–J03.X, and J06.X)
Lower respiratory tract disorders	
Hospitalization for pneumonia	Hospitalization with a principal diagnosis of ICD-10 code (J12.X–J18.X)[3]
Hospitalization for LRI	Hospitalization with a principal diagnosis of ICD-10 code (J20.X–J22.X)
Hospitalization for asthma	Hospitalization with a principal diagnosis of ICD-10 code (J45.X–J46.X)[4]
Gastrointestinal disorders	
Hospitalization for gastroenteritis	Hospitalization with a principal diagnosis of ICD-10 code (A00.X–A09.X)
Irritable bowel syndrome	Hospital visits more than one time with ICD-10 codes (K58.X and R10.X) or one or more hospital visits with ICD-10 codes (K59.1, K52.2, K52.8, or K59.0) after 4 years of age[5, 6]
Neurologic and behavioral disorders	
Attention-deficit hyperactivity disorder	Diagnosis of ICD-10 code (F90.X) after 6 years of age[7]
Febrile convulsion	Diagnosis of ICD-10 code (R56.X)
Epilepsy	Diagnosis of ICD-10 code (G40.X–G41.X) with two or more prescriptions of antiepileptic drugs within 6 months[8]
Immunological disorders	
Kawasaki disorder	Hospitalization with ICD-10 code (M30.3) with prescription of intravenous immunoglobulin[9]
Idiopathic thrombocytopenic purpura	Diagnosis of ICD-10 code (D69.3)[10]
Cancer	Diagnosis of ICD-10 codes (C00.X–C97.X)[11]

Supplementary Table S2. Definitions of perinatal conditions and comorbidities

**Disease**	**Definitions**
Perinatal comorbidities, n (%)	
Fetus and newborn affected by maternal conditions	Diagnosis of ICD-10 code P00.X–P04.X
Birth trauma	Diagnosis of ICD-10 code P10.X–P15.X
Respiratory and cardiovascular disorders specific to the perinatal period	Diagnosis of ICD-10 code P20.X–P29.X
Infections specific to the perinatal period	Diagnosis of ICD-10 code P35.X–P39.X
Hemorrhagic and hematological disorders of fetus and newborn	Diagnosis of ICD-10 code P50.X–P61.X
Transitory endocrine and metabolic disorders specific to fetus and newborn	Diagnosis of ICD-10 code P70.X–P74.X
Digestive system disorders of fetus and newborn	Diagnosis of ICD-10 code P75.X–P78.X
Conditions involving the integument and temperature regulation of fetus and newborn	Diagnosis of ICD-10 code P80.X–P83.X
Comorbidities, n (%)	
Hospitalization for wheezing	Hospitalization with a principal diagnosis of ICD-10 code J45.X, J46.X, or J21.X
Atopic dermatitis	Diagnosis of ICD-10 code L20.X and two prescriptions of topical steroid
Food allergy	Diagnosis of ICD-10 code L23.6, L24.6, L25.4, L27.2, T78.0, T78.1, or K52.2 before the first 1 year of life

Supplementary Table S3. Results of proLCA function

Number of clusters	AIC	BIC	G2	χ^2	n of estimated parameters	Residual degrees of freedom	Maximum log likelihood	Entropy
2	2,112,834	2,113,487	36,348	59,528	63	236,309	-1,056,354	
3	2,083,888	2,084,874	13,530	16,165	95	236,277	-1,041,849	
4	2,077,217	2,077,704	7,888	7,918	127	236,245	-1,038,561	
5	2,075,898	2,077,547	6,347	6,281	159	236,213	-1,037,790	
6	2,074,637	2,076,618	5,033	4,985	191	236,181	-1,037,127	
7	2,073,335	2,075,648	3,710	3,603	223	236,169	-1,036,444	
8	2,072,537	2,075,182	2,836	2,716	255	236,117	-1,036,013	
9	2,072,445	2,075,422	2,711	2,606	287	236,085	-1,035,935	
10	2,071,856	2,075,165	1,958	1,859	319	236,053	-1,035,609	

Abbreviations: LCA, latent cluster analysis; AIC, Akaike Information Criterion; BIC, Bayesian Information Criterion; G2, likelihood ratio/deviance statistic; $\chi2$, the chi-square statistic.

Supplementary Table S4. Risk of childhood diseases according to the cluster of feeding type during infancy[1]

	Cluster 1 (Reference, n=116,372)			Cluster 2 (n=108,189)				Cluster 3 (n=11,811)			
**Diseases**	**n of event**	**Accumulated n, 1,000 PY**	**IR/1,000PY**	**n of event**	**Accumulated n, 1,000 PY**	**IR/1,000PY**	**aHR[2] (95% CI)**	**n of event**	**Accumulated n, 1,000 PY**	**IR/1,000PY**	**aHR[2] (95% CI)**
Upper respiratory tract disorders											
Adenotonsillectomy	3,873	1,160.6	3.34	3,981	1,078.4	3.69	1.040 (0.993–1.088)	396	117.8	3.36	0.965 (0.868–1.074)
Chronic otitis media	912	1,169.0	0.78	819	1,087.0	0.75	0.955 (0.867–1.052)	88	118.7	0.74	0.950 (0.759–1.189)
Hospitalization for croup	943	1,167.4	0.81	935	1,085.0	0.86	1.049 (0.956–1.151)	127	118.3	1.07	1.204 (0.991–1.462)
Hospitalization for URI	3,496	1,154.6	3.03	3,497	1,072.4	3.26	1.058 (1.009–1.110)	491	116.5	4.21	1.307 (1.186–1.440)
Lower respiratory tract disorders											
Hospitalization for pneumonia	4,258	1,152.2	3.70	4,258	1,069.0	3.98	1.072 (1.026–1.119)	606	115.9	5.23	1.349 (1.236–1.472)
Hospitalization for LRI	124	1,172.8	0.11	146	1,090.2	0.13	1.180 (0.923–1.508)	13	119.0	0.11	0.810 (0.425–1.544)
Hospitalization for asthma	22	1,173.3	0.02	32	1,090.7	0.03	1.493 (0.864–2.581)	2	119.1	0.02	-
Gastrointestinal disorders											
Hospitalization for gastroenteritis	9,168	1,129.0	8.12	9,048	1,046.3	8.65	1.037 (1.007–1.069)	1,167	113.3	10.30	1.165 (1.093–1.240)
Irritable bowel syndrome	21,564	1,095.0	19.69	20,614	1,015.7	20.30	1.021 (0.944–1.093)	2,226	111.1	20.04	1.001 (0.967–1.041)
Neurologic and behavioral disorders											
Attention-deficit hyperactivity disorder	2,084	1,166.9	1.79	1,993	1,084.8	1.84	1.349 (1.189–1.530)	313	118.1	2.65	1.381 (1.218–1.566)
Febrile convulsion	2,912	1,155.2	2.52	2,908	1,073.0	2.71	1.069 (1.014–1.127)	367	116.8	3.14	1.222 (1.093-1.366)
Epilepsy	527	1,171.1	0.45	577	1,088.2	0.53	1.179 (1.044–1.331)	78	118.7	0.66	1.430 (1.120–1.827)
Immunological disorders											
Kawasaki disorder	1,234	1,161.2	1.06	1,159	1,079.4	1.07	1.009 (0.930–1.095)	102	118.1	0.86	0.820 (0.666–1.010)
Idiopathic thrombocytopenic purpura	126	1,172.8	0.11	126	1,090.3	0.12	1.051 (0.817–1.353)	13	119.0	0.11	0.957 (0.528–1.735)
Cancer	192	1172.2	0.16	165	1,089.9	0.15	0.910 (0.734–1.125)	19	119.0	0.16	0.973 (0.599–1.580)

Abbreviations, n, number; PY, person-year; IR, incidence rate; aHR, adjusted hazard ratio; CI, confidence interval; ICU, intensive care unit; URI, upper respiratory infection; LRI, lower respiratory infection.

[1]Children were classified based on their feeding patterns in infancy by using polytomous variable latent class analysis.

[2]Adjusted hazard ratios and their 95% confidence intervals were calculated using a Cox proportional hazard model, with adjustments for sex, region at birth, economic status, birthweight, body weight at 4–6 and 9–12 months of age, head circumference at 4–6 months of age, perinatal comorbidities, and comorbidities.

Supplemenatary Table S5. Associations of overweight/obesity at 6 years of age with the feeding pattern in infancy

	Total N	N of events (%)	RR	95% CI	Adjusted RR	95% CI
Overweight (z-score >1.036)						
Cluster 1	39,433	12,834 (32.55)	Ref	-	Ref	-
Cluster 2	37,097	12,242 (33.00)	1.026	1.001–1.052	1.067	1.040–1.094
Cluster 3	4,362	1,548 (35.49)	1.188	1.188–1.127	1.178	1.116–1.243
Obesity (z-score >1.0645)						
Cluster 1	39,433	4,989 (12.65)	Ref	-	Ref	-
Cluster 2	37,097	4,825 (13.01)	1.040	1.000–1.082	1.086	1.043–1.131
Cluster 3	4,362	671 (15.38)	1.325	1.223–1.436	1.273	1.172–1.383

Figure S1. The model's fit indices of the Latent Class Models

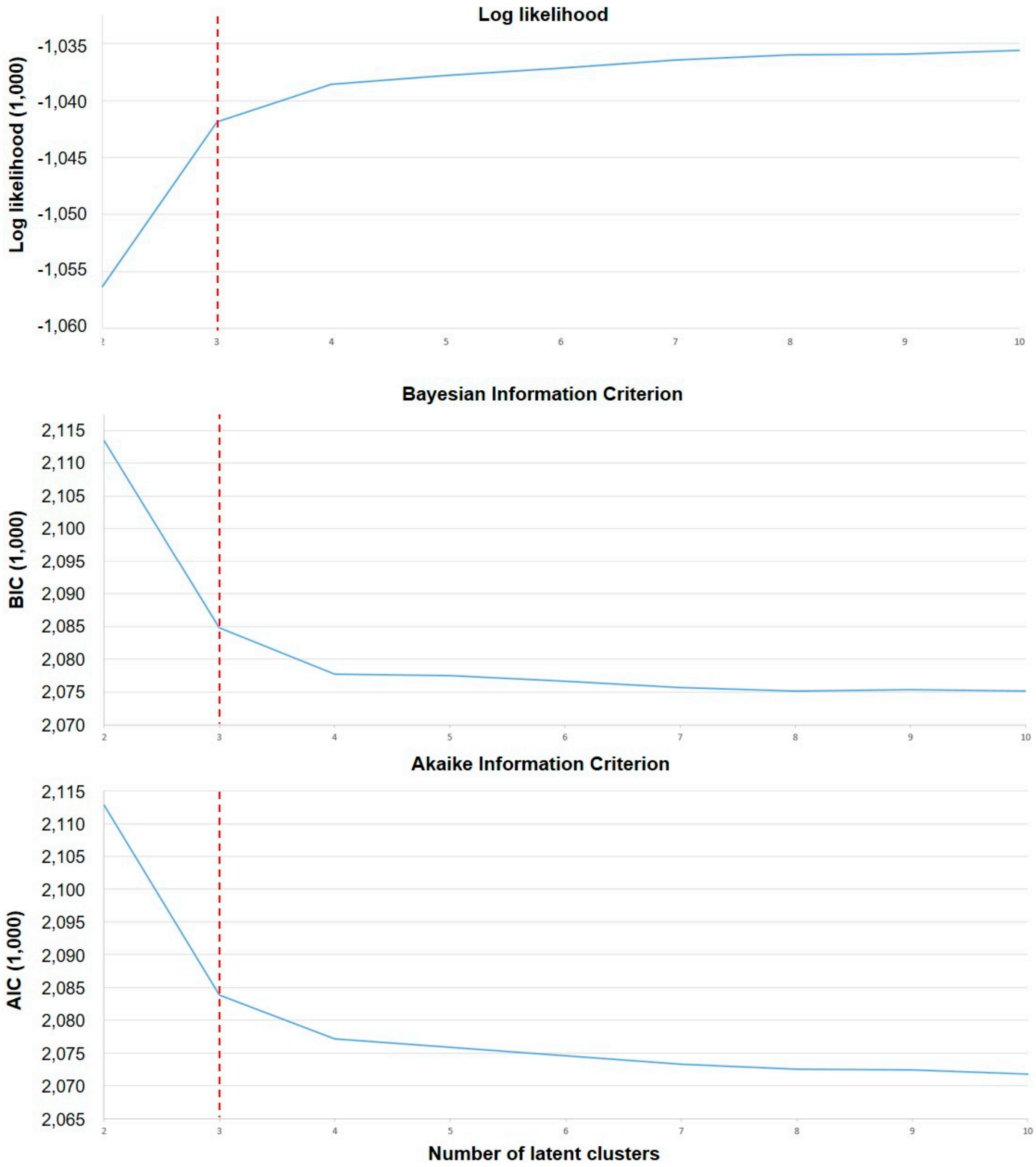

AIC, Akaike Information Criterion; BIC, Bayesian Information Criterion

References

1. Ha EK, Lee SW, Kim JH, et al. Changes in childhood growth after adenotonsillectomy: a population-based cohort study. *Sleep Medicine*. 2022;89:114-121.
2. Lin S-C, Lin H-W, Chiang B-L. Association of croup with asthma in children: A cohort study. *Medicine*. 2017;96(35).
3. Chang Y-P, Yang C-J, Hu K-F, et al. Risk factors for pneumonia among patients with Parkinson's disease: a Taiwan nationwide population-based study. *Neuropsychiatric Disease and Treatment*. 2016;12:1037.
4. Kim JH, Ha EK, Lee SW, et al. Growth pattern during early infancy, body mass index during childhood and childhood asthma. *Clinical & Experimental Allergy*. 2022.
5. Kim JH, Lee SW, Kwon Y, et al. Infantile Colic and the Subsequent Development of the Irritable Bowel Syndrome. *Journal of Neurogastroenterology and Motility*. 2022;28(4):618-629.
6. Goff SL, Feld A, Andrade SE, et al. Administrative data used to identify patients with irritable bowel syndrome. *Journal of clinical epidemiology*. 2008;61(6):617-621.
7. Kim JH, Yi YY, Ha EK, et al. Neurodevelopment at 6 years of age in children with atopic dermatitis. *Allergology International*. 2022.
8. Chiang K-L, Cheng C-Y. Prevalence and neuro-psychiatric comorbidities of pediatric epilepsy in Taiwan: a national population-based study. *Epilepsy research*. 2014;108(8):1451-1460.
9. Kwak JH, Ha EK, Kim JH, et al. Association of Familial History of Diabetes, Hypertension, Dyslipidemia, Stroke, or Myocardial Infarction With Risk of Kawasaki Disease. *Journal of the American Heart Association*. 2022;11(12):e023840.
10. Hung GY, Lee CY, Yen HJ, Lin LY, Horng JL. Incidence of immune thrombocytopenia in Taiwan: a nationwide population-based study. *Transfusion*. 2018;58(11):2712-2719.
11. Lin H-C, Chao Y-H, Wu K-H, et al. Increased risk of herpes zoster in children with cancer: A nationwide population-based cohort study. *Medicine*. 2016;95(30).
